# Pharmacokinetic and Pharmacodynamic Study of Folic Acid-Modified Chitosan–Stearic Acid Nanomicelles Loaded with Tetrandrine for Rheumatoid Arthritis

**DOI:** 10.3390/pharmaceutics17020169

**Published:** 2025-01-27

**Authors:** Shuai Ma, Fei Xue, Lan Yang, Long Chen, Pei Liu, Jinhua Chang, Ruxing Wang

**Affiliations:** 1 Hebei Province Key Laboratory of Research and Development for Chinese Materia Medica, Institute of Chinese Materia Medica, Chengde Medical University, Chengde 067000, China; 2 Basic Medical Institute, Chengde Medical University, Chengde 067000, China

**Keywords:** tetrandrine, nanomicelles, pharmacokinetics, pharmacodynamics

## Abstract

Background: Rheumatoid arthritis (RA) is a chronic autoimmune disease, and it is currently incurable. Tetrandrine (TET) has an obvious curative effect with therapeutic efficacy on RA, but its use is limited due to its poor water-solubility and bioavailability. Therefore, TET-loaded nanomicelles modified with chitosan, stearic acid, and folic acid (FCST) was prepared in the study, and the pharmacokinetics and pharmacodynamics were studied. Methods: The plasma concentrations of FCST and TET were measured by the PLC-MS/MS method at different times, and the pharmacokinetic parameters were calculated. A collagen-induced arthritis (CIA) model was established with rats. On the 16th day after the first immunization, 50 rats were randomized into five groups with 10 rats in each group according to the arthritis score. The drugs were administered by intraperitoneal injection for 30 days. The swelling degree and joint score of the rats were tested during each administration. In addition, the pro-inflammatory factors IL-1β, IL-6, IL-17, and TNF-α in the serum of the rats were tested by an ELISA kit, and their joints were examined by histopathology. Results: Pharmacokinetic studies showed that the AUC_0–72h_ of FCST was 1.93 times that of TET. FCST demonstrated higher bioavailability compared to TET (*p* < 0.05). Pharmacodynamic studies demonstrated that FCST had significant anti-inflammatory effects, and its anti-inflammatory activity was stronger compared to the same dose of TET, as evidenced by measuring toe thickness and observing toe appearance. It significantly reduced the expression of IL-1, IL-6, IL-17, and TNF-α in rats with rheumatoid arthritis (*p* < 0.05). Conclusions: FCST can significantly improve bioavailability and has a significant therapeutic effect on rheumatoid arthritis.

## 1. Introduction

Rheumatoid arthritis (RA) is a chronic autoimmune disease characterized by symptoms such as joint pain, tumescence, and deformation, and it leads to high rates of disability [1], causing significant suffering for patients, so the prevention and treatment of RA have become problems that need to be solved [2,3]. Currently, clinical drug treatments for RA are generally focused on relieving inflammation and controlling disease progression, such as through the use of nonsteroidal anti-inflammatory drugs, glucocorticoids, and anti-rheumatic drugs. Most of the drugs have low bioavailability because they are insoluble in water [4].

Nanotechnology has recently gained more attention from researchers, providing new opportunities for some major diseases [5,6,7]. Studies have shown that nanodrug delivery systems have certain advantages in diagnosing and treating RA [8,9,10]. Nanocarriers can deliver drugs to the sites of inflammation compared to traditional drugs selectively, thereby improving drug efficacy without affecting non-synovial tissues.

Various carriers are commonly used for drug delivery, including polymer micelles (PMs), liposomes, polymer nanoparticles, magnetic nanoparticles, etc. [11,12]. The carriers can achieve active targeting through surface group modification [13,14,15,16]. The hydrophobic core of the PMs can load water-insoluble drugs, increasing their solubility. The hydrophilic shell forms a protective barrier through hydration, thus reducing the adsorption of proteins during blood circulation, limiting clearance by the reticuloendothelial system and extending the half-life of drugs. The particle size of PMs is usually small and has a narrow distribution, which can be controlled by adjusting the length of hydrophilic and hydrophobic blocks. A characteristic of PMs is their clear structure and easy modification. In polymer synthesis, multifunctional PMs are designed by adding ligands (small molecules, antibody fragments, peptides [17,18,19], etc.) and chemical groups to improve the concentration of PMs at inflammation sites and their internalization by specific cells. PMs also respond to certain stimuli (enzymes, pH, anoxia, light, heat, etc.), facilitating the rapid release of drugs in specific areas [20,21].

Tetrandrine (TET), a natural isoquinoline alkaloid, is the primary active component of *Stephania tetrandra S. Moore* [22]. Pharmacological studies have confirmed TET’s multi-faceted effects, including anti-inflammatory, anti-tumor, and antihypertensive properties [23,24]. The impact of TET on IL-1β, IL-6, and TNF-α in the joint cavity and peripheral blood of CIA rats was investigated. It was found that after treatment with TET, the decreased levels of these cytokines in the joint cavity fluid and serum indicate that TET can treat CIA by regulating these changes [25]. These outcomes underscore TET’s ability to modulate critical inflammatory mediators, presenting a promising avenue for CIA treatment and its potential development as an anti-rheumatoid arthritis medication. Thus, we believe it can be developed as a drug with anti-rheumatoid arthritis effects. However, TET’s hydrophobic nature poses challenges to its bioavailability, limiting its clinical application. Therefore, to improve its targeting and bioavailability, FCSTs were prepared, and their pharmacokinetics and pharmacodynamics were investigated to provide a reference for their application. Currently, FCST has not been reported in the literature.

## 2. Materials and Methods

### 2.1. Materials and Reagents

Tetrandrine (TET, >98.0% purity, Shanchuan Biotechnology Co., Ltd., Xi’an, China), internal standard (IS) theophylline (TON > 98.0% purity, Pfeiffer Biotechnology Co., Ltd., Chengdu, China), chitosan (CS, molecular weight 2000, Haidebe Marine Biological Engineering Co., Ltd., Jinan, China), stearic acid (SA, Fuchen Chemical Reagent Factory, Tianjin, China), 1-ethyl-3-(dimethylaminopropyl) carbodiimide (EDC, Gill Biochemical Co., Ltd., Shanghai, China), folic acid (F, Rujian Biotechnology Co., Ltd., Shanghai, China), N-hydroxysuccinimide (NHS, Aladdin Reagent Co., Ltd., Shanghai, China), N,N′-dicyclohexylcarbodiimide (DCC, Aladdin Reagent Co., Ltd., Shanghai, China), and dimethyl sulfoxide (DMSO, Bailingway Technology Co., Ltd., Beijing, China) were used.

### 2.2. Animals

Sixty Wistar male rats (220 ± 10 g, license number SCXK2019-0008, Vital River Laboratory Animal Technology Co., Ltd., Beijing, China) were kept in a controlled breeding environment with free access to food and water.

### 2.3. Preparation of FCST

A total of 0.5 g CS was dissolved in 40 mL deionized water and was kept in a water bath at 80 °C. After dissolving 0.45 g SA in 60 mL anhydrous ethanol, EDC was added to SA solution and stirred at 60 °C for 30 min in a water bath. SA solution was added dropwise to the solution of CS slowly and was subsequently stirred magnetically at 400 r/min in the 80 °C water bath for 6 h. The solution was dialyzed by ultrapure water for 48 h in a dialysis tube after the reaction. The product was lyophilized to obtain a white lyophilized powder of CS, which was rinsed three times with anhydrous ethanol and dried at 60 °C to obtain CS-SA powder. A total of 40 mg folic acid (F) was dissolved in 10 mL DMSO, then DCC and NHS (1:1:2) were added. Then, 100 µL of triethylamine (TEA) was added and the mixture was stirred magnetically at 25 °C for 12 h with nitrogen. After the reaction, dicyclohexylurea (DCU) was removed by filtration. About 207 mg CS-SA was dissolved in 20 mL water, to which the filtered active folic acid ester was slowly added dropwise and stirred magnetically for 24 h, protected from light. After the reaction, the solution was dialyzed for 48 h through a regenerated cellulose dialysis bag to remove the incompletely reacted folic acid, and the water was changed four times a day to obtain FCS solution. After freeze-drying, a light-yellow FCS lyophilized powder was obtained.

A certain amount of TET and FCS (mass ratio was 1:2) were weighed precisely, mixed thoroughly with an appropriate amount of deionized water, and sonicated by the probe for some time (set at a certain power, working for 2 s, stopping for 3 s, under an ice bath) to obtain FCST suspension, and FCST lyophilized powder was obtained by freeze-drying.

The encapsulation efficiency of FCST was 98.86 ± 0.30%, the drug loading was 28.57 ± 0.34%, the average particle size was 227.0 ± 9.4, the average nm PDI was 0.42 ± 0.04, and the zeta potential was 12.6 ± 2.3 mV [26]. The critical micelle concentration (CMC) was 11.24 ± 0.31 μg/mL.

### 2.4. Infrared Spectral Scanning (FT-IR)

The CS, SA, CS-SA, and FCS samples were weighed at 2.0 mg each and dried at 100 °C for 2 h. The samples were mixed with potassium bromide, ground into powder, and pressed into sheets. The infrared absorption spectra of each sample in the range of 4000–400 cm^−1^ were measured and analyzed by infrared spectra.

### 2.5. Pharmacokinetic Study

Twelve male rats (220 ± 10 g) were divided into 2 groups randomly, and they were fasting for 12 h before administration and drinking water freely. FCST solution and TET were intraperitoneally injected at a dose of 2.7 mg/kg (12). At 0.083, 0.16, 0.25, 0.50, 1, 2, 4, 6, 8, 12, 24, 36, 48, and 72 h after the end of drug administration, 0.2 mL of blood was collected from orbit and put in heparinized EP tubes. After centrifugation for 10 min at 13,000 r/min, the plasma was separated and frozen at −80 °C.

### 2.6. UPLC-MS/MS Method

Agilent Acquity UPLC liquid chromatography included a diode array detector, injection manager, and binary pump. The chromatographic separations were carried out on an Agilent UPLC C_18_ column (2.1 × 50 mm, 1.7 μm; Agilent Corporation, Santa Clara, CA, USA) with a guard column. The mobile phase consisted of acetonitrile-10 mM ammonium acetate and 0.02% formic acid (90:10, *v*/*v*). The experimental conditions were as follows: the column temperature was 25 °C, the injection volume was 1 μL, the flow rate was 0.3 mL/min, and the running time was 3 min. The mass spectrometer was Provideda Q-Trap 5500 triple quadruple mass spectrometer (AB SCIEX, Boston, MA, USA) with a turbo ion spray electrospray ionization (ESI) source for the sample analysis.

MS/MS parameters were set as follows: ion spray voltage, +5500 V; curtain gas, 35 psi; temperature, 450 °C; ion source gas 1, 40 psi; ion source gas 2, 40 psi; the interface heater was on; and the collision gas was medium. The mass transitions were TET, *m*/*z* 623.5→381.2; theophylline, *m*/*z* 181.1→124.1; the declustering voltage of TET was 120 V, the collision voltage was 52.80 eV; the declustering voltage of theophylline was 98.62 V, and the collision voltage was 27.96 eV.

The standard curve of TET was linear (r^2^ > 0.99), and the lower limit of quantification was 0.1 ng/mL within the concentration range of 2–1000 ng/mL. The limit of detection was the concentration when the S/N was equal to 3. RSDs of the inter-day and intra-day accuracy were below 6.0%. The extraction recovery of TET was 96.09% and matrix effect was 95.67%, which were 100.59% and 105.98% for IS [27].

### 2.7. Pharmacodynamic Study

The rats were fed for 1 week and adapted to the environment. Ten rats were selected as the blank group randomly, and the rest were anesthetized with 20% urethan at 1.0 g/kg intraperitoneally, the hair on the back and tail root of the rats was cut off, and 0.25 mL of emulsion (10 mL bovine type II collagen [2 mg/mL] mixed with isopyknic CFA) was injected intradermally at multiple points throughout. The same method was used with a successful molding 7 days later; at 14 d after the first injection, the AI score of every rat was assessed [28].

To investigate the therapeutic effect of FCST on post-modeling Wistar rats, rats with successful CIA model establishment were divided into 5 groups (each group of 10 rats) randomly. Sixteen days after the initial immunization, the rats were injected intraperitoneally with FCST solution as high-, medium-, and low-dose groups (equivalent doses of TET were 5.40 mg/kg, 2.70 mg/kg, and 1.35 mg/kg), TET solution as TET group (equivalent dose of TET was 2.70 mg/kg), and an isopyknic saline of CIA rats and normal rats as the model control group and negative control group, respectively, for 30 d of continuous injection.

### 2.8. Measurement of Toe Thickness in Rats

The diameter of the left hind foot and ankle of each rat was measured using vernier calipers at the completion of treatment (14th day after initial immunization). The changes in AI values of the rats were monitored and recorded [29].

Pathological section observation of ankle joint bursa in rats was performed.

The experimental rats were killed 30 days after administration. The skin was peeled 0.5 cm above and below the ankle of the hind limb, and the joint tissues were taken, rinsed with normal saline, fixed with 4% formaldehyde for 2 days, embedded in paraffin, sliced, and then stained by HE and observed under an optical microscope [30].

### 2.9. Effect on Histopathology of Spleen in RA Rats

The spleen of the rat was removed and cleaned with PBS to remove the blood drops and the fat. It was fixed with neutral formaldehyde, paraffin-embedded tissue wax blocks were prepared, and routine sections (5 μm) were made and HE staining was performed. The morphologic and pathological changes in the spleen were observed with an optical microscope and photographed.

### 2.10. Cytokine Determination

The inflammatory factors (IL-1, IL-6, IL-17, TNF-α) in plasma were detected by an ELISA kit. A total of 1 mL of blood was gathered from the orbital venous plexus 20 days after administration. The anticoagulant was 1% heparin sodium solution. The samples were centrifuged for 10 min at 5000 rpm, the upper serum samples were absorbed, and the corresponding inflammatory factors were detected [31].

### 2.11. Statistical Analysis

The experimental data were presented as mean ± SD. Statistical analysis was performed by a pharmacokinetic and pharmacodynamic study using an one-way ANOVA. *p* < 0.05 was known as significant statistical differences.

## 3. Results

### 3.1. FT-IR Analysis

**Figure 1:**
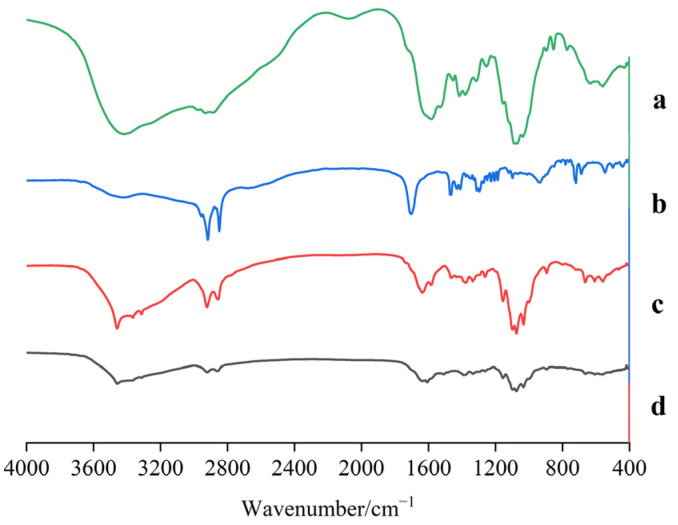
Infrared spectrum of CS (a), SA (b), CS-SA (c), and FCS (d).

Figure 1 shows that in the infrared spectrum of CS, the stretching vibration of the hydroxyl group and amino group produced an overlapping broad peak of 3416.03 cm^−1^, the carbonyl group in SA moved from 1704 cm^−1^ to 1636 cm^−1^ of the amide bond carbonyl in CS-SA, and the stretching vibration peak of the secondary amine at 3313 cm^−1^ appeared at the same time. This indicates that the condensation reaction of CS and SA occurred and CS-SA was formed. In the FCS infrared spectrum, in addition to the amide bond carbonyl absorption peak of CS-SA, it was found that 1608 cm^−1^ was the carbonyl absorption peak of the amide bond formed by the reaction of the carboxyl group in F with the amino group on the CS in CS-SA, and the characteristic absorption peak of the benzene ring appeared at 1509 cm^−1^. It was proved that FA had been successfully incorporated into the structure of CS-SA and FCS was obtained.

### 3.2. Pharmacokinetic Results

**Figure 2:**
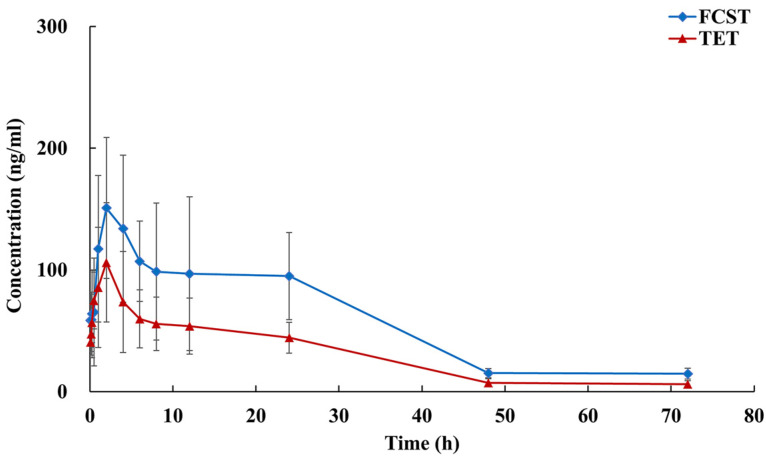
Blood concentration–time profiles for FCST (mean ± SD, *n* = 6).

According to the analysis in Figure 2 and Table 1, the area under the drug–time curve (AUC) of the FCST group was significantly larger than that of the TET group, being 1.93 times higher. The peak drug concentration (C_max_) of the FCST group was larger than that of the TET. In addition, the time to peak (T_max_) of the FCST group was significantly longer than that of the TET. The clearance (CL) of the FCST group was significantly lower than that of the TET. The results of this experiment suggested that the pharmacokinetic parameters of FCST changed compared with TET significantly.

### 3.3. Pharmacodynamic Results

**Figure 3:**
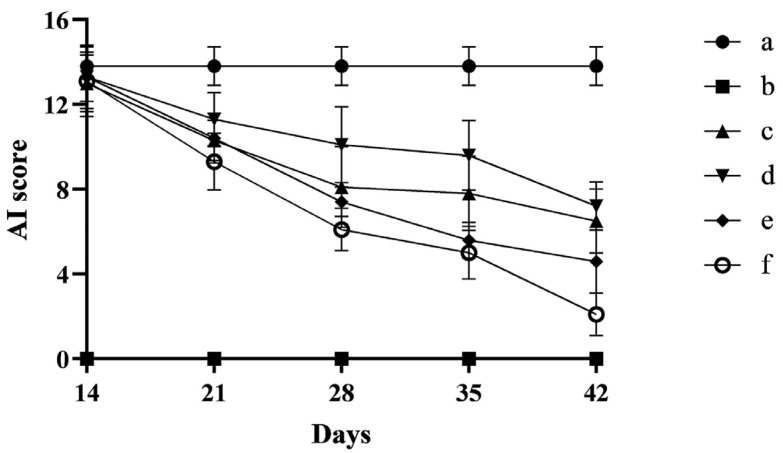
Variation in AI scores during treatment in rats (a. blank group, b. model group, c. TET group, d. FCST group (1.35 mg/kg), e. FCST group (2.70 mg/kg), f. FCST group (5.40 mg/kg)) (mean ± SD, *n* = 8).

In this study, after the start of modeling, the success of the CIA rat model was evaluated using the arthritis index score. After 14 days of immunization, it was indicated that all rats were successfully modeled when the sum of the arthritis index of the extremities exceeded six. The score results are shown in Figure 3.

**Figure 4:**
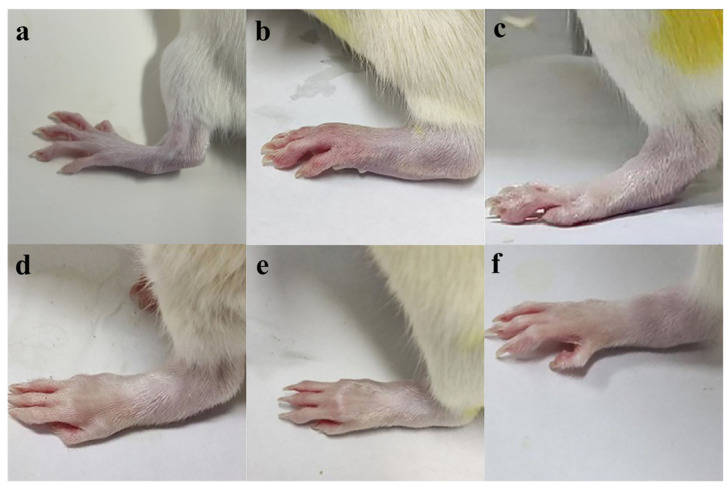
Toe thickness of rats with arthritis (mean ± SD, *n* = 8) ((**a**) blank group, (**b**) model group, (**c**) TET group, (**d**) FCST group (1.35 mg/kg), (**e**) FCST group (2.70 mg/kg), (**f**) FCST group (5.40 mg/kg)).

In the experiments, an emulsion of bovine type II collagen mixed with an isopyknic of CFA was used to induce an arthritis model for CIA rats to assess the role of FCST on rheumatoid arthritis. The rats of successful modeling appeared to have redness and swelling of the foot, with symptoms significantly worsening over time. After successful modeling, the CIA rats were administered daily intraperitoneal injections. To objectively evaluate the therapeutic effect of each dosing group, the anti-inflammatory effect of FCST was evaluated using changes in AI scores as well as rat toe thickness. Figure 4 showed that the model group remained essentially unchanged during the treatment period, and all treatment groups exhibited therapeutic effects on CIA rats, with the most significant in the high-concentration FCST treatment group (*p* < 0.01). Compared with the TET group, the same concentration of FCST reduced the arthritis score of CIA rats significantly (*p* < 0.05).

### 3.4. Toe Thickness of Rat Results

**Figure 5:**
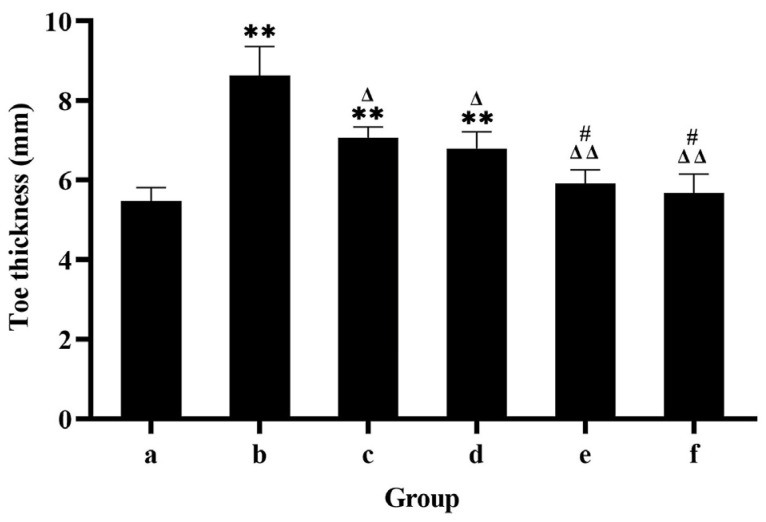
Appearance of the rat’s toes at the end of treatment (a. blank group, b. model group, c. TET group, d. FCST group (1.35 mg/kg), e. FCST group (2.70 mg/kg), f. FCST group (5.40 mg/kg)). Note: comparison with blank group, ** *p* < 0.01; compared with model group, ^Δ^ *p* < 0.05, ^ΔΔ^ *p* < 0.01; compared with TET, ^#^ *p* < 0.05.

Figure 5 shows that the toe thickness was (5.48 ± 0.34) mm in the blank group and (8.63 ± 0.74) mm in the model group. The swelling of the CIA rats decreased with increasing concentrations of FCST, with the most significant reduction in toe thickness observed in the high-dose group significantly (*p* < 0.01). Compared to the model group group, intraperitoneal injection of the each dose of FCST significantly reduced toe thickness and improved joint swelling in CIA rats (*p* < 0.01), which was also statistically significant. At the end of administration, the toes of CIA rats in the model group exhibited the most severe redness and swelling, and the toe thickness did not change during the treatment period. With increasing concentrations of FCST, the redness and swelling of the toes gradually subsided, and the toe thickness also decreased to some extent. Compared with the TET group, the toes of CIA rats in the each FCST concentration group showed slight relief, and their toe thickness also decreased slightly (*p* < 0.05).

### 3.5. Pathological Section of Ankle Joint Bursa in Rats

**Figure 6:**
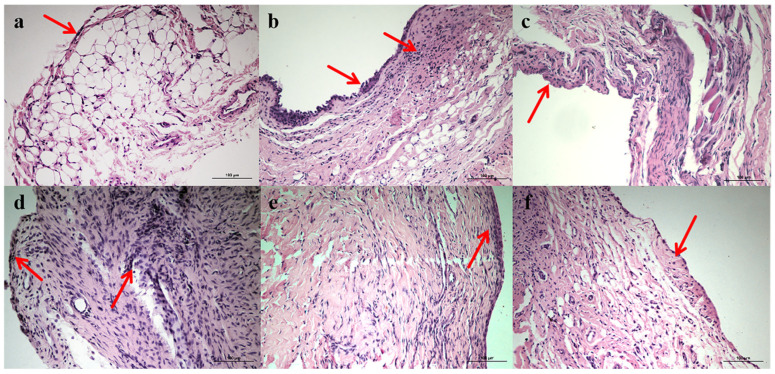
Effects of FCST on ankle joint histopathology of RA rats ((**a**) blank group, (**b**) model group, (**c**) TET group (**d**) FCST group (1.35 mg/kg), (**e**) FCST group (2.70 mg/kg), (**f**) FCST group (5.40 mg/kg)), (×400), arrows represent synovial thickening in each group.

The pathological sections of joint tissue of each group are shown in Figure 6 under a light microscope. Thirty days after administration, the synovial epithelial cells were arranged regularly with 1–2 layers, and there was no inflammatory cell infiltration in the subepithelial tissue in the normal group. In the model group, synovial epithelial cells proliferated, the number increased, the number of layers thickened, and there were dilated and congested capillaries and inflammatory cells infiltrated in the subepithelial tissues. Compared with the model group, the synovial morphology of rats in the FCST group had a better therapeutic effect, which can effectively relieve synovial tissue hyperplasia and decrease inflammatory cell infiltration. The therapeutic effect of a high dose was better than that of the medium- and low-dose groups. Compared with the FCST group, there was no significant therapeutic effect in the TET group; the synovial tissue thickened and inflammatory cells infiltrated.

**Figure 7:**
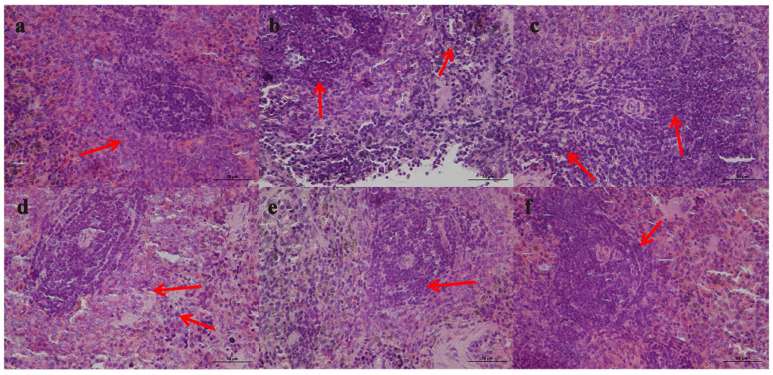
Effects of FCST on spleen histopathology of RA rats ((**a**) blank group, (**b**) model group, (**c**) TET group (**d**) FCST group (1.35 mg/kg), (**e**) FCST group (2.70 mg/kg), (**f**) FCST group (5.40 mg/kg)), (×400), arrows represent the pathological changes of the spleen in each group.

Pathological section of histopathology of spleen in RA rats:

The spleen of normal rats consists of white and red pulp, where the white pulp is traversed by a central artery and surrounded by a marginal area. In the model rats, the white pulp of the spleen was significantly enlarged, the red pulp was congested, inflammatory cells infiltrated, and a germinal center was formed. In addition, hyaline and pyknosis of the splenic trabeculae were also observed. Compared with the model group, the FCST groups had better therapeutic effects on the spleen structure, which could effectively alleviate the enlargement of white pulp, the congestion of red pulp, the infiltration of inflammatory cells, the hyalinization of splenic trabeculae, the nuclear pyknosis, and the number of germinal centers was significantly reduced. The treatment effect of the high-dose group was better than that of the medium-dose group and low-dose group. There was no significant therapeutic effect in the TET group compared with the model group. There were still enlarged white pulp, congestion of red pulp, inflammatory cell infiltration, transparency of splenic trabecula, nuclear pyknosis, and germinal centers, as shown in Figure 7.

### 3.6. Cytokine Determination Results

**Figure 8:**
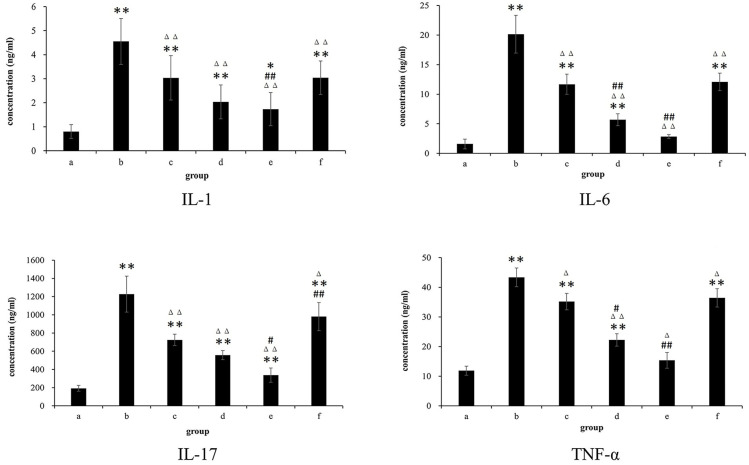
The expression on serum levels of IL-1, IL-6, IL-17, and TNF-α in rats with arthritis (mean ± SD, *n* = 8) (a. blank group, b. model group, c. TET group, d. FCST group (1.35 mg/kg), e. FCST group (2.70 mg/kg), f. FCST group (5.40 mg/kg)). Note: comparison with blank group, * *p* < 0.05, ** *p* < 0.01; compared with model group, ^Δ^ *p* < 0.05, ^ΔΔ^ *p* < 0.01; compared with TET, ^#^ *p* < 0.05, ^##^ *p* < 0.01.

In the study on the influence of the factors of protein expression, it was found that the contents of IL-1, IL-6, IL-17, and TNF-α in the serum of rats in the model group, TET, and each FCST concentration group were higher than that in the normal control group significantly. Compared with the model group, the contents of the above-mentioned factors in the serum of rats in TET and each FCST concentration group were lower than that in the model group significantly, showing that each drug administration group had therapeutic effects on model rats, as shown in Figure 8.

## 4. Discussion

As an autoimmune disease, RA is mainly manifested as joint dysfunction. According to surveys, the number of people with RA is about 1% of the global population [32]. The incidence rate of RA has shown an increasing trend year by year, with a high disability rate and a low remission rate, which threaten human health seriously. At present, the clinical treatment for RA mainly involves the use of non-steroidal anti-inflammatory drugs, glucocorticoids, and other medications, which have significant side effects on the liver, kidneys, and blood system [33]. Therefore, it is particularly important to develop anti-RA drugs with significant efficacy and minimal side effects.

Chitosan (CS) is a biodegradable polymer with good biocompatibility. The amino group on CS chemically reacts with the carboxyl group on stearic acid to form an amide bond, producing CS-SA. After dehydration, folic acid (F) forms an active ester, which is then aminated. The activated carboxyl part of folic acid (F) is covalently linked to the amino group on CS to obtain FCS [34]. When used as a carrier, it can enhance the release of drugs and reduce their toxic side effects [35].

Studies have shown that folate receptors are generally overexpressed in inflammatory cells [36,37], and FRβ is a true marker of the synovial macrophage subpopulation. There is a large amount of FRβ expression on macrophages triggered by inflammatory stimulation and activated macrophages in the inflammatory joints of RA patients. Thus, targeted therapy for FRβ has been extensively studied [38,39]. TET loaded with FCS can improve the water solubility of TET, and its physical and chemical properties are relatively stable.

In this study, according to the results of pharmacokinetic experiments, the relative bioavailability of FCST was 193% compared with that of TET, indicating that FCST could improve bioavailability. In addition, compared with the TET group, CL in the FCST group was significantly reduced and T_max_ was slightly longer than in the TET group, indicating that the FCST group had a longer circulation time before being completely cleared by the liver and kidney and had higher blood drug concentration. The above experimental results prove that FCST could prolong the blood circulation in vivo and increase the drug concentration in the blood.

The spleen is an important immune organ of the body. The pathological study found that the boundary between red and white pulp of normal rat spleen was clear, and there was almost no germinal center. The amount of white pulp in the spleens of the CIA rat model group was significantly increased, the limit between red pulp and white pulp was blurred, there was a lot of congestion in the red pulp, and there was the formation of germinal centers. After FCST administration, the white pulp decreased. With the increase in drug dose, the boundary between red pulp and white pulp became clearer, the number of germinal centers decreased, and the congestion of red pulp was alleviated. The above morphological changes in the spleen indicate that FCST can alleviate spleen inflammation in RA [40,41].

Cytokines are proteins secreted or expressed by activated immune cells, and their main biological function is to mediate and regulate immune and inflammatory responses [42]. It has been reported that cytokines that promote synovial autoimmunity and induce cartilage destruction play an important role in RA development [43]. IL-1, IL-6, IL-17, and TNF-α are important pro-inflammatory factors in the pathogenesis of RA, which keep the body in a chronic inflammatory state. The factors such as TNF-α and IL-1β in the peripheral blood of RA patients are significantly elevated [44]. In addition, IL-6, as a pleiotropic inflammatory factor, is involved in joint inflammation and destruction, mediating extra-articular systemic responses [45]. IL-17 contributes to the pathogenesis of RA by promoting synovial cell growth and joint injury and can synergistically increase the pro-inflammatory response with other factors such as TNF-α [46]. IL-1β, a key inflammatory factor in RA, can trigger a range of cellular responses, including downstream gene induction and inflammatory responses. The study showed that FCST significantly inhibited the secretion of serum pro-inflammatory cytokines such as IL-6, IL-1β, TNF-α, and IL-17.

## 5. Conclusions

In this study, pharmacodynamic studies showed that FCST had significant anti-inflammatory effects, and its anti-inflammatory activity was stronger than that of the same dose of TET, as measured by the AI score, toe thickness, observations of toe appearance in rats, and the pathological section of histopathology of the spleen in RA rats. FCST showed a good therapeutic effect on the CIA rats. The results of this experiment suggested that the pharmacokinetics of FCST changed compared with TET significantly and FCST can achieve a good release effect. The study provided an idea for the treatment of RA. Thus, FCST is a promising formulation.

## Figures and Tables

**Table 1:** Pharmacokinetic parameters of TET and FCST in rats after drug delivery (mean ± SD, *n* = 6. * *p* < 0.05, ** *p* < 0.01).

Parameter	TET	FCST
AUC_0–t_/µg·L^−1^·h	2161.22 ± 529.28	4171.38 ± 1659.13 *
t_max_/h	2.08 ± 1.56	3.33 ± 0.03 *
t_1/2_/h	19.06 ± 5.69	23.22 ± 4.17 *
CL/L·h^−1^·kg^−1^	1.19 ± 0.25	0.65 ± 0.21 **
C_max_/µg·L^−1^	114.46 ± 45.88	159.41 ± 55.55 **

Each value represents the mean of six independent determinations ± SD, *: significant differences vs. TET (*p* < 0.05), **: significant differences vs. TET (*p* < 0.01).

## Data Availability

Data are contained within the article.
